# Plasma Lactoferrin Levels Positively Correlate with Insulin Resistance despite an Inverse Association with Total Adiposity in Lean and Severely Obese Patients

**DOI:** 10.1371/journal.pone.0166138

**Published:** 2016-11-30

**Authors:** Sylvain Mayeur, Alain Veilleux, Yves Pouliot, Benoît Lamarche, Jean-François Beaulieu, Frédéric S. Hould, Denis Richard, André Tchernof, Emile Levy

**Affiliations:** 1 Research Centre CHU Sainte-Justine, Université de Montréal, Montreal, Quebec, Canada; 2 Institute of Nutrition and Functional Foods (INAF), Université Laval, Quebec, Quebec, Canada; 3 Department of Anatomy and Cellular Biology, Faculty of Medicine and Health Sciences, Université de Sherbrooke, Sherbrooke, Quebec, Canada; 4 Institut universitaire de cardiologie et de pneumologie de Québec, Quebec, Canada; 5 Department of Nutrition, Université de Montréal, Montreal, Quebec, Canada; John Hopkins University School of Medicine, UNITED STATES

## Abstract

**Context:**

Lactoferrin (Lf) is an important protein found on mucosal surfaces, within neutrophils and various cells, and in biological fluids. It displays multiple functions, including iron-binding as well as antimicrobial, immunomodulatory and anti-inflammatory activities. Although Lf ingestion has been suggested to cause adiposity reduction in murine models and humans, its relationship with insulin resistance (IR) has not been studied thoroughly.

**Objective:**

To establish the association between circulating Lf levels, glucose status and blood lipid/lipoprotein profile.

**Methods:**

Two independent cohorts were examined: lean to moderately obese women admitted for gynecological surgery (n = 53) and severely obese subjects undergoing biliopancreatic diversion (n = 62).

**Results:**

Although body mass index (BMI) and total body fat mass were negatively associated with Lf, IR (assessed by the HOMA-IR index) was positively and independently associated with plasma Lf concentrations of the first cohort of lean to moderately obese women. These observations were validated in the second cohort in view of the positive correlation between plasma Lf concentrations and the HOMA-IR index, but without a significant association with the body mass index (BMI) of severely obese subjects. In subsamples of severely obese subjects matched for sex, age and BMI, but with either relatively low (1.89 ± 0.73) or high (13.77 ± 8.81) IR states (according to HOMA-IR), higher plasma Lf levels were noted in insulin-resistant *vs* insulin-sensitive subjects (*P*<0.05). Finally, Lf levels were significantly higher in lean to moderately obese women than in severely obese subjects (*P*<0.05).

**Conclusion:**

Our findings revealed that plasma Lf levels are strongly associated with IR independently of total adiposity, which suggests an intriguing Lf regulation mechanism in conditions of obesity and IR.

## Introduction

Obesity is an alarming problem worldwide in view of its close association with a plethora of comorbidities, resulting in a heavy socioeconomic burden [1]. It increases the incidence of traditional cardiovascular risk factors like insulin resistance (IR), which is a prime aetiological factor for hypertension, dyslipidemia and type 2 diabetes (T2D) [1]. The growing obesity prevalence may reflect the limited potential role of public health initiatives and current pharmacological treatments for contending obesity-related cardiometabolic disorders although this issue remains a matter of debate. Therefore, there is an urgent need to discover not only new powerful drugs, but also functional foods, which may synergistically help curb the epidemic of obesity-related metabolic diseases [2, 3]. Currently, various research groups advocate the use of milk-derived proteins, including Lf, as preventive and curative tools for alleviation of complex metabolic diseases [4, 5].

Lf is present in high concentrations in human and bovine milk, as well as in lower amounts in exocrine secretions (i.e. saliva, tears, semen, vaginal fluids, gastrointestinal fluids) and cells (i.e. neutrophils, leukocytes, enterocytes and adipocytes) [6–10]. This iron-binding glycoprotein possesses antimicrobial activity and it also improves immune defences against pathogenic bacteria and viruses [11]. In addition, Lf was shown to lower inflammation, oxidative stress (OxS) and apoptosis, which are key mechanisms involved in the progression of various cardiometabolic disorders [12–14] while few reports have emphasized its anti-adipogenic actions [15, 16]. Accordingly, Moreno-Navarrete et al. proposed that endogenous Lf biosynthesis is essential to achieve adequate adipogenesis [17]. In this context, circulating Lf levels were significantly reduced in subjects with obesity [18], and Lf ingestion (over an 8-week period) decreased visceral adiposity in men [4]. On the other hand, Kim et al. reported that Lf concentrations are associated with the high risk of obesity-related phenotypes in children, a finding quite divergent from the observations in adults [19]. So far, the associations among endogenous Lf, obesity and accompanying metabolic disorders have not been exhaustively examined. In addition, there was no attempt to evaluate the correlation between Lf and obesity while covering a wide range of body weight and IR states, as most of the cohorts from previous studies consisted of overweight subjects with no appropriate lean or healthy controls [18, 20]. Moreover, no thorough investigations have scrutinized the independent impact of adiposity and IR on circulating Lf levels. Finally, the contribution of the small intestine to plasma Lf levels in the presence of IR and T2D have not been explored even if the gut is clearly endowed with the expression of Lf protein and its specific receptors [7, 21]. Hence, the aim of the present work was to establish the association between circulating Lf levels, glucose status and blood lipid/lipoprotein profile while correcting for adiposity indices.

## Materials and Methods

### Subjects and tissue sampling

The first study cohort included 53 lean to obese women (aged from 39 to 62 years) who were recruited through the elective surgery schedule of the Gynaecology Unit of Laval University Medical Center [22, 23]. Women underwent abdominal gynaecological surgery for total (n = 50) or subtotal (n = 3) abdominal hysterectomies, some with salpingo-oophorectomy of one (n = 11) or two ovaries (n = 23). Reasons for surgery included one or more of the following factors: myoma (n = 36), menorrhagia (n = 33), fibroids (n = 18), ovarian cyst (n = 16), endometriosis (n = 11), dysmenorrhoea (n = 11), endometrial hyperplasia (n = 5), pelvic adhesions (n = 4), polyps (n = 3), pain (n = 3), adenomyosis (n = 2), uterine bleeding (n = 1), and/or ovarian thecoma (n = 1). The exclusion criteria include cancer, coronary heart diseases, T2D, thyroid disorders, Cushing’s syndrome, major weight changes in the last 6 months as well as medications such as beta-blockers, ACE inhibitors and statins. Using menstrual history questionnaires, medical files and blood FSH levels, we have identified 36 pre- or perimenopausal women, 11 postmenopausal and 6 women receiving hormonal replacement therapy.

The second study cohort included 62 non diabetic and 10 severely obese subjects with T2D (BMI ≥ 40 kg/m^2^) undergoing bariatric surgery (biliopancreatic diversion) at the Quebec Heart and Lung Institute, Laval University [24]. Identification of non-diabetic and T2D subjects was performed according to the diagnosis available in the medical file (before the surgery). Twenty of the non-diabetic subjects (10 women and 10 men), for which we had small intestine samples, were assigned to two groups matched for sex, age (*±* 10 years) and BMI (± 5 units), but with either relatively low (< 3) or high (> 7) IR according to their HOMA-IR index [24]. Patients in the non-diabetic group did not have any treatment with insulin, hypoglycaemic agents and cholesterol- or triglyceride-lowering agents. As previously mentioned, ten subjects previously diagnosed with T2D were also recruited. These subjects received anti-diabetic treatments, including one or more of the following drug classes: metformin (n = 9), thiazolidinedione (n = 4), insulin analogue (n = 4), sulfonylurea (n = 3) and dipeptidyl peptidase-4 inhibitor (n = 1). Eight of these subjects also received lipid-lowering drugs, including atorvastatin (n = 5) and rosuvastatin (n = 3). During the surgery, duodenum specimens were obtained from severely obese patients included in this second cohort. Intestinal specimens were immediately frozen in liquid nitrogen and stored at –80°C.

The project was approved by the ethics committees of Laval University Medical Center, Quebec, Canada), Quebec Heart and Lung Institute (Laval University, Quebec City, Canada) and Sainte-Justine Research Center (University of Montreal, Montreal, Canada). Written informed consent was obtained from all subjects.

### Total adiposity and body fat distribution measurements

BMI was measured on the morning of the surgery for subjects recruited in the two study cohorts. In the first group of 53 lean to moderately obese women, total body fat mass was measured using dual-energy X-ray absorptiometry, and abdominal body fat distribution was obtained at the L4-L5 vertebrae level using the Light-Speed 1.1 CT-scanner (General Electric Medical Systems, Milwaukee, USA) within a few days before the surgery [24, 25].

### Lipid profile and glucose homeostasis

Overnight fasting blood samples were drawn on the morning of the surgery. Lipid profile was measured as previously described [25]. Glucose was assessed using the glucose oxidase method and insulin was quantified with the ultrasensitive insulin assay employing Access^®^ immunoassay system (Beckman Coulter, Brea, USA). The HOMA-IR index was calculated using the following formula: fasting insulin (μU/mL) x fasting glucose (mmol/L) ÷ 22.5 [26]. Monitoring of the fasting plasma glucose (just before surgery) revealed that 6 women from the gynecology unit (first cohort) as well as 13 very obese subjects from the second cohort [excluding subjects (n = 10) recruited with a previous T2D diagnosis] had a slightly impaired fasting glycaemia (6.1 mmol/L). In the absence of a thorough T2D medical diagnosis, these subjects were included in the non-diabetic group.

### Plasma Lf

Plasma Lf concentrations were determined in plasma (after a night of fasting) using an ELISA kit (Assaypro EL2011-1, St.-Charles, USA) that is highly specific to human Lf. The intra- and inter-assay coefficients of variation were 4.8 and 7.3%, respectively. The cross-reactivity was less than 1% for bovine Lf.

### Cell culture and treatments

Intestinal Caco-2/15 cells were cultured at sub-confluence in MEM supplemented with 5% fetal bovine serum, 1% non-essential amino acids and 1% Penicillin/Streptomycin (Gibco, Grand Island, USA) as previously described [27]. Cells were cultured for 21 days post-confluence, which represents an appropriate period to reach full differentiation and to study OxS and inflammation [28]. Cells were serum starved for 18 h before being incubated with or without lipopolysaccharide (LPS) (150 μg/mL) for 24 h at 37°C.

### Lf mRNA expression

Total RNA was isolated using the RNeasy extraction kit (Qiagen, Valencia, USA) and complementary DNA was generated using the Superscript first strand synthesis system (Invitrogen, Carlsbad, USA). Real-time cDNA amplification was performed in duplicate using SYBR Green with the 7500 Real-Time PCR System (Applied Biosystems, Foster, USA) for 40 cycles. Target gene amplifications were normalized using expression levels of ATP synthase 5 subunit O (ATP5O), which was not associated with variables examined in this study. Results were validated using a second housekeeping gene: hypoxanthine phosphoribosyl transferase 1 (HPRT1). The relative mRNA fold changes between groups were calculated using the 2^−ΔΔCt^ method. The sequences of the primers for Lf were 5'-GAACCGTACTTCAGCTACTCTG-'3 and 5'-CTCATACTCGTCCCTTTCAGC-'3, and for ATP5O were 5'-GCGATGCTTCAGTACCTCTG-'3 and 5'- TGGCATAGCGACCTTCAATA-'3. As one sample displayed an amplification problem, the corresponding matched subjects were excluded from the present analysis (n = 9)

### Western blot analysis

Proteins were denatured in sample buffer and separated on a 10% SDS-PAGE and electroblotted into Hybond nitrocellulose membranes (Amersham, Baie D’Urfé, Canada). The following antibodies were used for immunoblotting: 1:2000 anti-human Lf (78 kDa; Santa Cruz Biotechnology, USA), 1:40000 anti-β-actin (42 kDa; Sigma, USA) and 1/10000 horseradish peroxidase-conjugated secondary antibodies (Jackson Laboratory, USA), The β-actin protein expression was determined to confirm equal loading.

### Statistical analyses

Differences in parameters between groups were tested using paired t-test and one-way ANOVA. Pearson correlation coefficients were computed to quantify associations between plasma Lf levels and anthropometric or biochemical parameters. Adjustment for sex, age and BMI was performed using the generalized linear model. Impact of menopausal status on these associations was also assessed. Variables that were not normally distributed based on a significant Shapiro-Wilk test (*P*≤0.05) were log_10_-transformed. Messenger RNA expression values (ΔΔCt) were compared using the Wilcoxon signed-rank test. Differences were considered statistically significant at *P*<0.05. Statistical analyses were performed with SAS software (SAS Institute, Cary, NC, USA).

## Results

Anthropometric and biochemical parameters of the first cohort composed of 53 lean to moderately obese women are shown in Table 1. These subjects were overweight according to their mean BMI of 26.7 kg/m^2^, which covered a wide range of adiposity values (17.2 to 39.4 kg/m^2^). Moreover, various body fat distribution patterns were noted in this cohort as shown by the variation of abdominal computed tomography measurements (Table 1). Mean plasma Lf levels in this first sample were 931 ± 387 ng/mL and ranged from 179 to 2023 ng/mL. As expected, IR was positively correlated to several adiposity variables, including BMI (r = 0.56; p<0.001, n = 53), fat mass (r = 0.54; p<0.001; n = 53) and visceral adipose tissue (r = 0.59; p<0.001; n = 52).

**Table 1:** Characteristics of the subjects included in the first study samples.

Variable	Mean ± SD	Range(min-max)
**Anthropometrics**		
Age (years)	47.5 ± 5.1	39.6–61.7
BMI (kg/m^2^)	26.7 ± 5.0	17.2–39.4
Fat mass (%)	34.9 ± 6.4	19.6–47.5
Total adipose tissue area (cm^2^)	427 ± 187	128–991
Subcutaneous adipose tissue area (cm^2^)	331 ± 146	94–758
Visceral adipose tissue area (cm^2^)	96.9 ± 46.8	33.7–232.4
**Glucose homeostasis**		
Glycemia (mmol/L)	5.59 ± 0.5	4.6–6.7
Insulin (pmol/L)	11.1 ± 5.5	3.4–27.6
HOMA-IR	2.8 ± 1.7	0.8–8.6
**Lipid profile**		
Total cholesterol (mmol/L)	4.8 ± 0.7	3.4–6.1
HDL cholesterol (mmol/L)	1.5 ± 0.4	0.8–2.9
LDL cholesterol (mmol/L)	2.7 ± 0.6	1.3–3.9
Triglycerides (mmol/L)	1.3 ± 0.8	0.5–2.7
Total cholesterol/HDL	3.5 ± 1.0	1.6–6.2
Lf (ng/mL)	931 ± 387	179–2023

**Fig 1:**
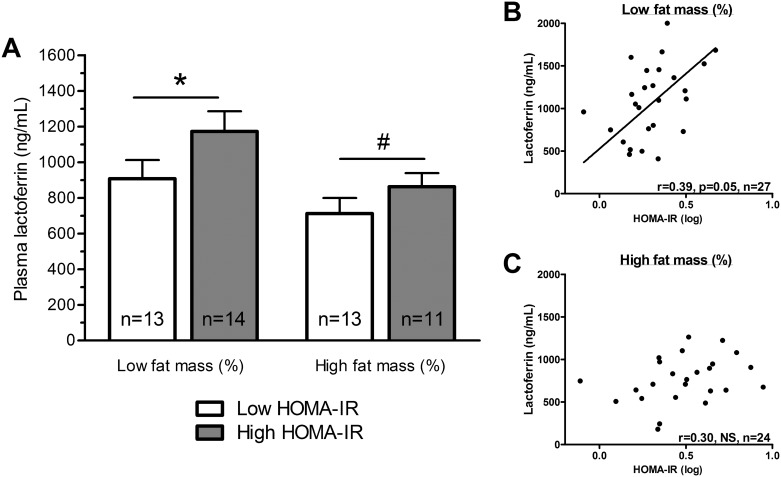
Plasma Lf levels in lean to moderately obese subjects. **(A)** Plasma Lf concentrations were assessed in lean to moderately obese women following stratification performed according to median values of body fat mass (median = 34.8%) and HOMA-IR index (low fat mass group: median = 1.8; high fat mass group: median = 3.1). Correlations were tested between plasma Lf concentrations and HOMA-IR in subjects with body fat mass (%) **(B)** lower or **(C)** higher than the median Pearson correlation coefficients of log-transformed variables. *P* values are shown on the graph: * *P*<0.05; # *P* = 0.1.

Data analysis revealed several negative associations between plasma Lf and adiposity indices (Table 2). In fact, subjects with elevated values of BMI, body fat mass or abdominal adiposity had reduced plasma Lf levels compared to leaner subjects. Nevertheless, subcutaneous and visceral adipose tissue areas showed similar correlation coefficients, suggesting that total adiposity rather than the specific fat distribution pattern was the main correlate of plasma Lf. No significant correlations were initially observed with glucose homoeostasis parameters. It is only when the specific contribution of adiposity and age was counterbalanced (using partial correlation analysis) that we noted a positive association between plasma Lf levels, fasting insulin concentrations and HOMA-IR index (Table 2). Exclusion of postmenopausal women and those receiving hormone replacement therapy did not significantly impact on the association of Lf with fasting insulin (0.30, n = 39, *P* = 0.06) and HOMA-IR index (0.31, n = 39, *P* = 0.05). This relationship is included in Fig 1 and was established when subjects were stratified into four subgroups according to median values of body fat mass and HOMA-IR index. Lean and insulin-sensitive women had lower Lf levels compared to their insulin-resistant counterparts (Fig 1A; *P*<0.05). A similar trend was observed in subjects with a high body fat mass (*P*<0.1) given that obese subjects had, on average, lower Lf levels compared to leaner subjects’ values (*P*<0.05) regardless of the HOMA-IR index (Fig 1A). Bivariate correlation analysis for each body fat mass subgroups revealed that plasma Lf was positively correlated with IR in relatively lean subjects but not in relatively obese subjects (Fig 1B and 1C). These observations suggest that (i) increased adiposity is associated with decreased Lf levels, and (ii) higher IR remains independently associated with higher Lf levels in leaner subjects only. Of note, in this first cohort, Lf levels were significantly associated with HDL3-cholesterol and not with the other blood lipid parameters (Table 2). Nevertheless, the latter association was not independent of total adiposity.

**Table 2:** Pearson correlation coefficients of plasma Lf and anthropometric with biochemical parameters in lean to moderately obese women.

Variables		Plasma Lf (ng/mL) unadjusted		Plasma Lf (ng/mL) adjusted for age and fat mass	
	N	r	*P* values	r	*P* values
**Anthropometrics**					
Age (years)	53	0.01	NS	-	-
BMI (kg/m^2^)	53	-0.30	0.03	0.05	NS
Fat mass (%)	53	-0.41	0.002	-	-
Total adipose tissue area (cm^2^)	53	-0.38	0.005	-0.20	NS
Subcutaneous adipose tissue area (cm^2^)	52	-0.37	0.01	-0.10	NS
Visceral adipose tissue area (cm^2^)	52	-0.31	0.03	-0.10	NS
**Glucose homeostasis**					
Glycemia (mmol/L)	53	-0.02	NS	0.15	NS
Insulin (pmol/L)	53	0.03	NS	0.39	0.01
HOMA-IR	53	0.06	NS	0.34	0.02
**Lipid profile**					
Total cholesterol (mmol/L)	52	-0.03	NS	-0.09	NS
HDL cholesterol (mmol/L)	52	0.17	NS	-0.06	NS
HDL2 cholesterol (mmol/L)	51	0.07	NS	-0.16	NS
HDL3 cholesterol (mmol/L)	51	0.29	0.04	0.10	NS
LDL cholesterol (mmol/L)	52	-0.03	NS	-0.06	NS
Triglycerides (mmol/L)	52	-0.16	NS	-0.03	NS
Total cholesterol/HDL	52	-0.14	NS	-0.01	NS
**Adipokines and inflammatory factors**					
Leptin (ng/mL)	49	-0.31	0.02	-0.09	NS
Resistin (ng/mL)	50	-0.25	0.08	-0.14	NS
Interleukin-6 (pg/mL)	49	-0.11	NS	-0.02	NS
Adiponectin (ng/mL)	49	0.01	NS	-0.04	NS

Pearson correlation coefficients were computed between plasma Lf and anthropometric or biochemical parameters in lean to moderately obese women of the first cohort. Partial correlation coefficients (r) and *P* values are provided for unadjusted analysis (left) and analysis adjusted for age and fat mass (right). Correlation coefficient of log10-transformed variables. BMI: Body mass index; NS: Non-significant;—: Not applicable.

The relation between Lf and some adipocyte inflammatory factors was also assessed in this cohort of lean to moderately obese subjects. Plasma Lf levels were negatively correlated to circulating leptin concentrations (-0.31, n = 49, *P*<0.02) with a trend correlation to resistin (-0.25, n = 50, *P* = 0.08). Noteworthy, these associations were not independent of fat mass and they appear to be only driven by the strong negative association between adiposity and plasma Lf levels. On the contrary, no associations were noted between Lf and circulating interleukin-6 or adiponectin concentrations (Table 2).

As our observation related to the positive correlation between Lf and IR appears discordant with previous reports [8, 18, 20], we attempted to assess this relationship in the second cohort comprising severely obese subjects (BMI range from 40.1 to 67.3) who were candidates for bariatric surgery (Table 3). We simultaneously evaluated the independent impact of IR and adiposity on plasma Lf levels. Mean plasma Lf levels in severely obese, nondiabetic subjects were 569 ± 235 ng/mL, ranging from 209 to 1280 ng/mL. Similar plasma Lf values (521 *±* 203 ng/mL, ranging from 298 to 963) were observed in severely obese subjects with T2D. However, lean to moderately obese subjects of the first cohort were characterized by high Lf concentrations (931 ± 387 ng/mL) that were more significantly (*P*<0.05) elevated than those of severely obese, nondiabetic subjects. No sexual dimorphism was observed in the Lf findings of the second cohort as men and women presented similar plasma Lf values (593 ± 202 *vs* 574 ± 251 ng/mL, NS).

**Table 3:** Characteristics of patients included in the second study sample.

Variables	Non-diabetic (n = 62)		Diabetic (n = 10)		*P* values
	Mean ± SD	Range (min-max)	Mean ± SD	Range (min-max)	
**Anthropometrics**					
Sex (women/men)	18/44		0/10		-
BMI (kg/m^2^)	53.3 ± 7.9	40.1–67.3	50.1 ± 8.0	40.8–62.5	NS
**Glucose homeostasis**					
Glycemia (mmol/L)	5.8 ± 0.7	4.4–6.9	8.6 ± 2.7	6.1–14.3	0.001
Insulin (pmol/L)	21.1 ± 15.8	4.1–90.2	27.1 ± 20.9	6.4–67.1	0.05
HOMA-IR	5.26 ± 3.94	1.05–22.5	9.9 ± 6.7	1.8–19.7	0.05
Glycated hemoglobin (%)	5.8 ± 0.43	5.0–7.3	7.5 ± 1.5	6.1–10.1	0.001
**Lipid profile**					
Cholesterol (mmol/L)	4.46 ± 1.02	2.93–6.69	4.04 ± 1.12	2.50–5.74	NS
Triglycerides (mmol/L)	1.56 ± 0.59	0.59–2.68	1.93 ± 1.99	0.70–7.22	NS
HDL cholesterol (mmol/L)	1.16 ± 0.25	0.69–1.89	1.13 ± 0.23	0.82–1.47	NS
LDL cholesterol (mmol/L)	2.68 ± 0.85	0.93–5.13	2.11 ± 0.85	0.91–3.28	0.05
Lf (ng/mL)	569 ± 235	209–1280	521 ± 203	298–963	NS

**Fig 2:**
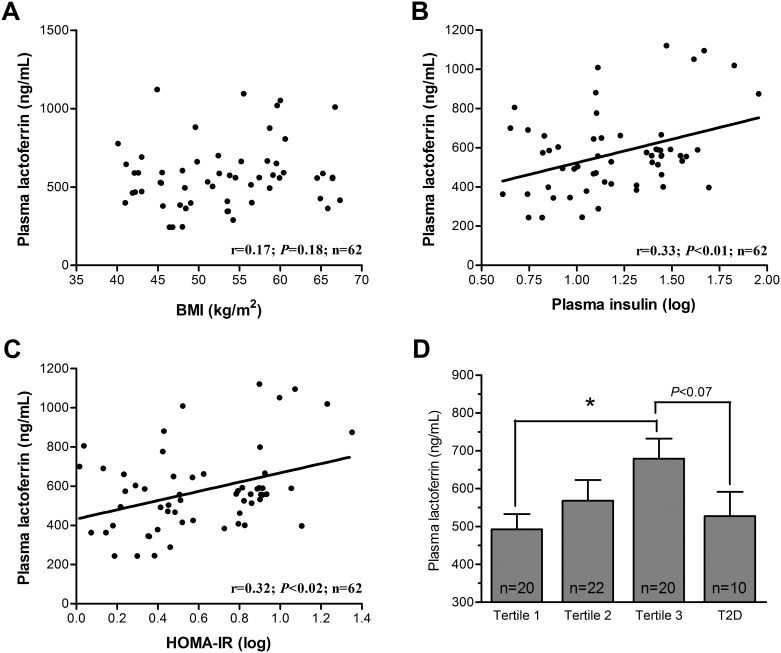
Plasma Lf levels in severely obese subjects. Correlations were tested between plasma Lf concentrations and **(A)** BMI, **(B)** insulin and **(C)** HOMA-IR in severely obese subjects without T2D. Pearson correlation coefficients of log-transformed variables and *P* values are shown in the graph (n = 62); **(D)** Plasma Lf concentrations in severely obese patients according to HOMA-IR tertiles and to the presence of T2D. * *P<*0.05.

Plasma Lf levels in the cohort of non-diabetic and severely obese subjects were not significantly related to age and BMI (Fig 2A), fasting glycaemia (data not shown) and plasma lipid parameters. Nevertheless, plasma Lf levels were positively and significantly associated with fasting insulin (Fig 2B) and HOMA-IR index (Fig 2C) in severely obese subjects. Then, partial correlation coefficients were computed for Lf and HOMA-IR following adjustment for sex, age and BMI (Table 4). Clearly, plasma Lf levels remained significantly associated with HOMA-IR (r = 0.31, n = 62, *P* = 0.03). This analysis was repeated in subgroups of relatively insulin-sensitive and insulin-resistant subjects, defined by a HOMA-IR index below or above the median (3.71). Interestingly, an independent association between plasma Lf levels and the HOMA-IR index was observed in subjects with relatively high IR values (r = 0.52, n = 31, *P* = 0.03), but not in those presenting lower IR values (r = 0.16, n = 31, NS). In fact, IR explained: (i) 31.1% of plasma Lf variance following adjustment for sex, age and BMI in the complete cohort of severely obese subjects and (ii) 52.2% in subjects with a relatively high IR states (Table 4).

**Table 4:** Adjusted partial correlation coefficients of plasma Lf concentration and HOMA-IR of severely obese women and men.

Study samples		Unadjusted		Adjusted for Sex		Adjusted for Sex. Age and BMI	
	N	r	*P* values	r	*P* values	r	*P* values
All subjects	62	32.4	0.02	31.1	0.02	31.1	0.03
Insulin-sensitive subjects (HOMA-IR<3.71)	31	12.6	NS	13.4	NS	16.4	NS
Insulin-resistant subjects (HOMA-IR>3.71)	31	44.1	0.02	43.4	0.02	52.2	0.03

Pearson correlation coefficients were computed for plasma Lf and HOMA-IR in severely obese patients. Partial correlation coefficient (r) and *P* values are provided for unadjusted analysis, analysis adjusted for sex, and analysis adjusted for sex, age and body fat mass. Correlation coefficients of log10-transformed variables are shown. BMI: Body mass index; NS: Non-significant.

Fig 2D displays the relationship between plasma Lf levels and IR following the stratification of subjects according to HOMA-IR index tertiles. Fig 2D also documents the same relationship in severely obese subjects with a previous T2D diagnosis. Plasma Lf levels (527 ± 203 ng/mL) of subjects with T2D were relatively similar to those of subjects in the first (493 ± 178 ng/mL) and the second (568 ± 256 ng/mL) tertiles but tended to be lower than those of the third tertile (679 ± 237 ng/mL) of non-diabetic subjects. These results suggest that plasma Lf levels are increased in subjects with an exaggerated IR state but not in those developing overt T2D and on pharmacological therapy for their conditions.

These aforementioned results were obtained using linear regression analysis in the second cohort of severely obese subjects. We challenged these conclusions using a subsample of severely obese subjects from the same cohort. Subjects were matched for sex, age and BMI but had either a low (<3; n = 10) or a high (>3; n = 10) HOMA-IR index. Anthropometric and biochemical characteristics of these subjects are shown in Table 5. By design, age and BMI were similar between the insulin-sensitive and the insulin-resistant subgroups but fasting insulin, HOMA-IR and glycated hemoglobin values were higher in the insulin-resistant subjects. The latter also presented higher triglyceride levels and lower HDL cholesterol concentrations compared to insulin-sensitive subjects (*P*<0.05). Plasma Lf levels were significantly (*P*<0.05) higher in insulin-resistant subjects (696.2 ± 205.6 ng/mL) than in insulin-sensitive subjects (555.1 ± 168.9 ng/mL). These differences were not related to differences in sex, age and BMI. They pointed out an independent relationship between IR and plasma Lf levels in severely obese subjects, which was coherent with the results obtained in the complete cohort of severely obese subjects and in the whole sample of lean to moderately obese women.

**Table 5:** Anthropometric and biochemical parameters as well as plasma Lf concentrations in a subsample of severely obese subjects matched for sex, age and BMI in high (n = 10) and low insulin sensitivity (n = 10) patients based on HOMA-IR.

Variables	Insulin-sensitive subjects (Mean ± SD; n = 10)	Insulin-resistant subjects (Mean ± SD; n = 10)	Paired t-test(*P* values)
**Anthropometrics**			
Sex (women/men)	5/5	5/5	-
BMI (kg/m^2^)	52.0 ± 7.9	51.5 ± 7.2	NS
**Glucose homeostasis**			
Glycemia (mmol/L)	5.44 ± 0.52	5.97 ± 0.65	NS
Insulin (pmol/L)	7.76 ± 3.02	51.43 ± 30.27	0.001
HOMA-IR	1.89 ± 0.73	13.77 ± 8.81	0.002
Glycated hemoglobin (%)	5.69 ± 0.002	5.92 ± 0.003	0.002
**Lipid profile (mmol/L)**			
Total cholesterol (mmol/L)	4.34 ± 0.89	4.25 ± 1.36	NS
Free cholesterol (mmol/L)	1.32 ± 0.24	1.29 ± 0.42	NS
Cholesterol ester (mmol/L)	2.90 ± 0.74	2.75 ± 0.94	NS
HDL cholesterol (mmol/L)	1.37 ± 0.32	1.11 ± 0.27	0.004
LDL cholesterol (mmol/L)	2.53 ± 0.79	2.65 ± 0.89	NS
Triglycerides (mmol/L)	1.14 ± 0.38	1.86 ± 0.47	0.0003
Totalcholesterol/HDL-cholesterol	3.47 ± 0.76	4.34 ± 0.74	0.005
LDL diameter (A)	254.4 ± 1.9	252.4 ± 1.6	0.01
Lf (ng/mL)	555.1 ± 168.9	696.2 ± 205.6	0.05

**Fig 3:**
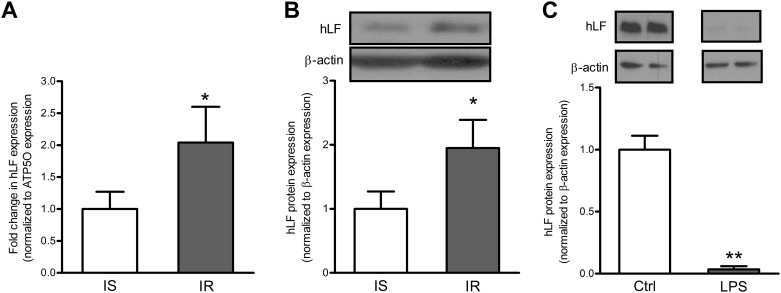
Intestinal Lf mRNA and protein expression in severely obese subjects and in Caco-2/15 cells. mRNA and protein levels of Lf were estimated in the intestine of insulin-sensitive and insulin-resistant obese subjects (n = 9 per group). The relative mRNA fold-changes between groups were calculated using the 2^−ΔΔCt^ method. mRNA data were normalized to ATP5O mRNA expression. Modulation of Lf protein following a 24-hour Caco-2/15 cell incubation with LPS (150 μg/mL). Protein expression values were normalized to β-actin protein expression. In B, samples were run on the same gel, but lanes were not contiguous. * *P*<0.05 vs. insulin-sensitive subjects, **p<0.001 vs. control cells.

Finally, we tested the possibility that the small intestine is implicated in these changes following its contribution to circulating Lf. Quantitative RT-PCR and Western blot analysis of intestinal Lf expression in severely obese subjects revealed that insulin-resistant individuals had increased Lf mRNA and protein levels compared to insulin-sensitive subjects (Fig 3A and 3B) matched for sex, age and BMI. The magnitude of intestinal Lf expression in the presence of an IR state, which was independent of adiposity differences, reflected Lf changes as a function of insulin sensitivity, but the opposite is also possible. The evaluation of obesity impact on intestinal Lf expression was limited by the availability of intestinal samples in leaner subjects. As obesity and inflammation generally induce IR, we tested the effect of bacterial LPS-mediated inflammation on intestinal Lf protein expression. Interestingly, the differentiated Caco-2/15 enterocytes produce significant levels of Lf, but the protein expression was totally blunted by the presence of LPS following a 24-hour incubation (Fig 3C). These results suggest that the intestine is also an important site of endogenous Lf production, but obesity-related factors (i.e. inflammation) and IR may strongly influence this condition.

## Discussion

Only few studies have so far attempted to define the associations among circulating Lf levels, adiposity markers and glucose homoeostasis [8, 18, 20, 29]. In addition, the reported results of these studies have only partially advanced our understanding of the role of Lf in cardiometabolic disorders. Our knowledge remains limited since most of the investigations failed to adequately discriminate the impact of adiposity from the effect of IR given the restricted range of adiposity values of their cohorts. Therefore, we carefully designed this study in order to assess the association between Lf and IR while correcting for the influence of adiposity on circulating Lf levels. It is important to stress that the present work is distinct from previous studies, e.g. the use of two independent cohorts covering a large range of adiposity values and IR degrees. We first determined plasma Lf levels in a cohort of lean to moderately obese women to estimate the influence of obesity and IR. This particular step allows us to point out that (i) plasma Lf levels were negatively associated with overall adiposity, and (ii) a compensatory increase in plasma Lf levels was recorded in response to high IR states. We could successfully confirm our former observations in a second cohort that was composed of severely obese subjects with various IR states. Comparison of the data from the two cohorts definitely indicated that Lf levels decreased as total adiposity increased, whereas IR was associated with elevated plasma Lf levels in any obesity condition.

Previous studies showed a negative correlation between plasma Lf levels and BMI or abdominal adiposity values in overweight subjects [18, 20]. Moreno-Navarette *et al*. reported that circulating Lf levels were lower in moderately obese subjects compared to overweight subjects [8]. This latter group also disclosed a positive correlation between plasma Lf levels and insulin sensitivity indexes assessed by a frequently sampled intravenous glucose tolerance test, a more accurate tool than HOMA-IR to assess the whole body insulin sensitivity [20]. The authors were not only able to document the positive relationship between decreased circulating Lf and IR (in contrast to our findings) but they also suggested that low Lf could play a role in inflammation-associated IR [20]. Our results are based on two independent cohorts that made it possible to reveal the independent correlation between Lf and IR when adjustments were made to correct for total adiposity. Moreover, our first study samples were focused only on women while Moreno-Navarrete et al. included only men in their population. Currently, it is difficult to ascertain the reasons for the ambiguities between the different studies [8, 18, 20] and additional efforts are definitely needed to clarify this important issue.

We also demonstrated that Lf levels in T2D subjects were comparable to those of insulin-sensitive individuals, at least to metabolically healthy subjects characterized by severe obesity. This observation is consistent with the previous report [29]. On the other hand, differences in plasma Lf levels were noted between insulin-resistant subjects and patients with T2D, probably due to the intensive pharmacological therapy provided to the latter. In this specific context, it is interesting to note that higher Lf concentrations in T2D subjects, which may be indicative of the unsuccessful therapy, have been previously associated with an augmented prospective risk of fatal ischemic heart disease [29].

On average, non-diabetic patients included in the second study cohort had the values of glycated hemoglobin slightly lower than those in diabetic subjects of the same cohort (5.8 vs. 7.2%). These non-diabetic subjects were not treated with insulin or hypoglycaemic agents during the long-term medical monitoring before surgery. However, subjects in the insulin-resistant group (Table 5) had normal glucose levels (5.97 mmol/L) with higher insulin levels that the insulin-sensitive group (51.43 vs. 7.76 pmol/L). Although these patients were severely obese, they successfully maintain their glucose levels in the physiological range. Consequently, glycated hemoglobin concentrations were only slightly increased in this subpopulation. Of note, non-diabetic patients receiving intensive anti-diabetic therapy were excluded from our study cohort as their insulin-resistance levels (HOMA-IR) were artificially reduced and could interfere with the interpretation of our results.

The precise mechanisms behind plasma Lf increase in the presence of IR have not been defined. Even if our observations are suggestive of a regulatory role of insulin in circulating Lf induction, we cannot exclude the impact of additional factors such as inflammation and OxS, which have the ability to deteriorate insulin sensitivity. On the other hand, Lf may exert a significant influence on insulin signalling and related functions, including: (i) improvement of Akt serine 473 phosphorylation; (ii) increase in GLUT4 and insulin receptor expression in mature adipocytes; (iii) enhancement of glucose disappearance rate; and (iv) reduction in inflammation and OxS [8, 16]. For now, we do not know if the positive correlations observed between Lf and IR represent a compensatory mechanism to alleviate IR. It is also unclear whether the augmented Lf levels in IR conditions denotes an attempt to preserve or maintain global Lf action despite the negative environment. To reconcile the contradictory findings—ours favouring the positive correlation between Lf and IR, and those of the literature favouring the association between Lf and insulin sensitivity- we speculate that Lf controls glucose homoeostasis under normal metabolic conditions, whereas insufficient or impaired Lf action may result in several metabolic disorders such as IR. Obviously, the intracellular pathways mediating the various actions of Lf on insulin signalling as well as the precise mechanisms by which Lf exerts its effects on cardiometabolic functions should be the subjects of future, thorough investigation.

Experimental data have shown that diet represents a significant source of exogenous Lf that can contribute to its availability in the gastrointestinal tract and blood circulation [30–32]. To distinguish between endogenous and exogenous Lf, we used a bioassay that specifically detects and measures endogenous Lf. In previous reports, some investigators normalized Lf concentrations to neutrophil counts and evidenced a decrease in Lf release from neutrophils in obese and insulin-resistant subjects [8, 18, 20]. It is important to recall that Lf is not only expressed in exocrine organs and polynuclear neutrophils, but also in various organs (i.e., adipose tissue, intestine and kidneys) [7–10]. The aim of the present study is to establish the association between circulating Lf levels and glucose homoeostasis while correcting for adiposity indices. Using the Lf-to-neutrophil ratio, our analysis would have been more focused on neutrophil functionality and their capacity to secrete Lf in insulin-resistant subjects, which is clearly not our goal. Thus, the report of all the results by reference to neutrophils would be inappropriate.

Obesity, IR and T2D are characterized by an inflammatory state [33, 34]. In this context, metabolic endotoxemia [higher plasma LPS levels] as a consequence of raised gut permeability has been proposed as the main cause for the inflammatory state [33, 34]. To approach the mechanisms of action, we assessed Lf expression in the intestine and impact of IR and bacterial LPS. We found that Lf was substantially expressed in the small intestine, but LPS-induced inflammation strongly suppresses its biogenesis. This highly suggests that the intestine may be an important and finely regulated contributor of circulating Lf. Interestingly, normal gut functions and intestinal barrier permeability were restored by exogenous Lf administration in LPS-challenged rats [33, 34] through various mechanisms (i), modulation of immune functions [35–37]; (ii) regulation of intestinal microbiota [35–37]; (iii) abolition of LPS-induced reactive oxygen species production [38]; (iv) inhibition of LPS binding to its receptors (i.e. CD14) [39]; and (v) reduction of OxS through L-selectin competition [40]. For now, although the intestine appears as an important source of endogenous Lf production, especially with increased Lf expression in IR (likely reflecting a protective effect), the fact that an inflammatory stimulus as LPS blunts the expression of Lf in the intestine is quite intriguing. We could assume at this point that Lf protective effects are offset in deleterious conditions.

Some experimental and clinical studies suggest that exogenous Lf administration may be a promising pharmaceutical agent to reduce fat accumulation. Dietary consumption of Lf during caloric restriction in mice improved weight loss and induced a strong decrease in adiposity and adipocyte size [41]. Similarly, bovine Lf administration to mice was shown to reduce mesenteric fat mass but failed to modulate body weight [5]. In human subjects, eight-week administration of enteric-coated Lf lowered total adiposity and visceral fat accumulation [4]. According to the authors, Lf may exert potent lipolytic activity. In this case, the decreased plasma Lf levels in obese subjects (noted in our study) might down-regulate adipose tissue lipolytic activity, thereby contributing to fat accumulation in the presence of a positive energy balance.

The use of two independent, well-characterized cohorts with widely variable adiposity distribution clearly represents strength of this study since it allowed us to establish important correlations between Lf and metabolic parameters. However, we must acknowledge some limitations. Intestinal samples were only available for the obese subjects, a shortcoming that did not allow comparisons between Lf levels of lean and obese individuals. Likewise, the cross-sectional design of this study was not fully optimal to definitely highlight the relationship among Lf, adiposity and IR in our cohorts. In fact, although our findings emphasize the negative and positive correlations with obesity and insulin resistance, respectively, additional longitudinal studies are required to conclude on the impact of Lf status on these cardiometabolic disorders.

Milk-derived proteins and peptides display biological activities towards the prevention of diseases. Recently, these bioactive components have gained interest because of their notable anti-hypertensive, antioxidant, anti-inflammatory and hypercholesterolaemia effects [4, 5]. A few studies, mainly in animals, suggested the potential benefits of exogenous Lf against lipid disorders, insulin signalling disturbances and inflammation [12–14]. The central message of this work, as mentioned above, relates to the independent relationship between concentrations of circulating Lf and insulin resistance. It is very probable that Lf plays a positive role in improving insulin sensitivity considering its impact on OxS and inflammation, two pathophysiological processes leading to adverse metabolic conditions such as insulin resistance. But in harmful cardiometabolic conditions, insufficient or impaired Lf action may occur as shown in our results. Is it an evident sign of compensation for metabolic disorders? Is it consequent to impairment of Lf receptors because of severe insulin resistance? For now, our studies can only be very speculative unless confirmative data are delivered by the results of further detailed investigations, which will allow the scientific and medical community to identify new forms of intervention or direct therapeutic strategies employing Lf to reduce metabolic disorder severity.

## Conclusions

The present investigation shows that plasma Lf levels are negatively associated with body adiposity. Plasma Lf concentrations have been found to positively correlate with insulin resistance states. Exaggerated intestinal Lf expression was also consistent with increased Lf levels in presence of systemic insulin resistance in obese subjects. Finally, the association between Lf and IR has been observed over a whole range of adiposity values. As Lf has been reported displaying anti-inflammatory, antioxidants and insulin-sensitizing functions, it is possible that the high plasma Lf levels, noted in cardiometabolic disorders, reflect insufficient or impaired Lf action or rather a potential to impede the sequence of events leading to IR development under obesity conditions.

## Abbreviations

BMI: body mass index
HDL: high-density lipoprotein
IR: insulin resistance
LDL: low-density lipoprotein
Lf: lactoferrin
T2D: type 2 diabetes
VLDL: very-low-density lipoprotein
